# TRPV1 Modulator Ameliorates Alzheimer-Like Amyloid-*β* Neuropathology via Akt/Gsk3*β*-Mediated Nrf2 Activation in the Neuro-2a/APP Cell Model

**DOI:** 10.1155/2022/1544244

**Published:** 2022-08-27

**Authors:** Xiufen Wang, Yaqi Bian, Clarence Tsun Ting Wong, Jia-Hong Lu, Simon Ming-Yuen Lee

**Affiliations:** ^1^State Key Laboratory of Quality Research in Chinese Medicine and Institute of Chinese Medical Sciences, University of Macau, Macao, China; ^2^Jiangsu Key Laboratory of Brain Disease and Bioinformation, Research Center for Biochemistry and Molecular Biology, Xuzhou Medical University, Xuzhou, China; ^3^Department of Applied Biology and Chemical Technology, The Hong Kong Polytechnic University, Hong Kong, China; ^4^Department of Pharmaceutical Sciences, Faculty of Health Sciences, University of Macau, Macao, China

## Abstract

Alzheimer's disease (AD) is a progressive and irreversible neurodegenerative disorder for which there is no effective therapeutic strategy. PcActx peptide from the transcriptome of zoantharian *Palythoa caribaeorum* has recently been identified and verified as a novel antagonist of transient receptor potential cation channel subfamily V member 1 (TRPV1). In the present study, we further investigated the neuroprotective potential of PcActx peptide and its underlying mechanism of action, in an N2a/APP cell model of AD. Both Western blot and RT-PCR analysis revealed that PcActx peptide markedly inhibited the production of amyloid-related proteins and the expression of BACE1, PSEN1, and PSEN2. Moreover, PcActx peptide notably attenuated the capsaicin-stimulated calcium response and prevented the phosphorylation of CaMKII and CaMKIV (calcium-mediated proteins) in N2a/APP cells. Further investigation indicated that PcActx peptide significantly suppressed ROS generation through Nrf2 activation, followed by enhanced NQO1 and HO-1 levels. In addition, PcActx peptide remarkably improved Akt phosphorylation at Ser 473 (active) and Gsk3*β* phosphorylation at Ser 9 (inactive), while pharmacological inhibition of the Akt/Gsk3*β* pathway significantly attenuated PcActx-induced Nrf2 activation and amyloid downregulation. In conclusion, PcActx peptide functions as a TRPV1 modulator of intercellular calcium homeostasis, prevents AD-like amyloid neuropathology via Akt/Gsk3*β*-mediated Nrf2 activation, and shows promise as an alternative therapeutic agent for AD.

## 1. Introduction

Alzheimer's disease (AD), which is one of the most prevalent neurodegenerative disorders, is clinically characterized by a progressive pattern of memory loss coupled with cognitive impairment [1, 2]. Studies have shown that AD contributes to 60-70% of all dementia cases [3, 4]. Senile plaques (SP) formed by extracellular accumulation of amyloid-*β* (A*β*) peptide and neurofibrillary tangles (NFTs) formed by intracellular deposition of hyperphosphorylated microtubule-associated tau protein are two critical pathological features of AD [5, 6]. Current pharmacological approaches for treating AD mostly target toxic A*β* and tau proteins; no effective treatments are available for halting or reversing the progression of the disease, although some therapies may be able to alleviate symptoms and improve patient quality of life [7]. A*β* peptides arise from sequential proteolytic cleavage of transmembrane amyloid precursor protein (APP) by *β*-secretase (BACE1) and the *γ*-secretase complex, which is called amyloidogenic pathway [8, 9]. Although the precise mechanisms underlying the A*β*-related pathogenesis of AD remain unclear, accumulating evidence suggests that calcium homeostasis perturbation and oxidative stress could drive the onset and progression of AD [10, 11]. A*β* deposition in the synapse could prompt the generation of reactive oxygen species (ROS) and disruption of calcium homeostasis, thereby triggering cell death and neurodegeneration [12]. Meanwhile, excessive intercellular calcium in turn enhances ROS generation, which leads to oxidative stress, mitochondrial dysfunction, and calcium signaling dysregulation [11]. Therefore, oxidative stress is both a cause and consequence of calcium homeostasis disruption. Nuclear factor erythroid 2-related factor 2 (Nrf2) is a key modulator of the endogenous antioxidative defense against oxidative stress. Under oxidative stress conditions, Nrf2 dissociates with Kelch-like ECH-associated protein 1 (Keap1), translocates to the nucleus, and then dimerizes with small Maf family proteins and binds to antioxidant response element (ARE) [13]. The interaction of Nrf2 and ARE promotes the transcription of downstream genes encoding phase II response antioxidative enzymes, such as heme oxygenase 1 (HO-1) and NAD(P)H quinone oxidoreductase (NQO1), to counteract oxidative stress [14]. Meanwhile, the protein kinase B (PKB or Akt)/glycogen synthase kinase 3*β* (Gsk3*β*) pathway is involved in the activation of Nrf2 by phosphorylation [15]. Since multiple reports have confirmed that activation of Nrf2 may prompt neuroprotective activity in neurodegenerative models, we assume that Akt/Gsk3*β*-mediated Nrf2 activation could be a target for preventing AD pathology [16–18].

Multiple protein and peptide drugs have been identified as having therapeutic potential for various neurological diseases [19–22]. Kunitz-type peptides can cross the blood-brain barrier (BBB) [23–25] and exhibit neuroprotective activity *in vivo* and *in vitro* [26–28]. PcActx peptide, a Kunitz-like peptide derived from zoantharian *Palythoa caribaeorum*, was the first transient receptor potential cation channel subfamily V member 1 (TRPV1) antagonist of zoantharian species discovered in our previous study [29]. PcActx peptide prevented pentylenetetrazol- (PTZ-) induced seizure-related behavior and inhibited ROS overproduction through regulating calcium and GABAergic-glutamatergic signaling [29]. TRPV1 is a calcium-permeable channel, and its activation impairs calcium homeostasis and induces the oxidative stress; this may contribute to cell death and neurodegeneration [30–32]. Kim *et al*. reported that the TRPV1 agonist capsaicin elicits cell death in neurons and microglia. TRPV1 activation increases the intercellular calcium concentration, triggers mitochondrial damage, and induces cytochrome c release and caspase-3 cleavage, which are all involved in AD pathogenesis. However, these effects are attenuated by the TRPV1 antagonist capsazepine and iodoresiniferatoxin (IRTX) [31, 32]. Furthermore, IRTX, a TRPV1 antagonist, can inhibit A*β*-induced ROS production and microglia priming, suggesting that the TRPV1 channel could be implicated in microglia-induced oxidative stress in AD [33]. In contrast to the inhibitory effect of TRPV1 antagonism on A*β* production, capsaicinoids (capsaicin and dihydrocapsaicin), as TRPV1 agonists, increase A*β* production by enhancing the expression of *β* and *γ*-secretase and inhibiting the A*β* degradation [34]. Calcium influx via the TRPV1 channel triggers Gsk3*β* activation, which stimulates the phosphorylation and ubiquitination of Nrf2 [35]. Consequently, modulation of TRPV1-mediated calcium homeostasis may be a novel target for therapeutic strategies for AD. In the present study, we aimed to investigate the neuroprotective potential and mechanism of action of PcActx peptide in the Neuro-2a cells stably transfected with the APP gene (N2a/APP cell), as an *in vitro* experimental model of AD.

## 2. Materials and Methods

### 2.1. Chemicals and Reagents

PcActx peptide was produced via an oxidative folding method after synthesizing the linear form, as described in our previous research [29]. Dulbecco's modified Eagle's medium (DMEM, Cat. No.: 12800017), fetal bovine serum (FBS, Cat. No.: 1805387), penicillin-streptomycin (PS, Cat. No.: 15140163), phosphate-buffered saline (PBS, Cat. No.: 21600010), and trypsin (Cat. No.: 25200072) were purchased from Gibco (Carlsbad, CA, USA). G418 (Cat. No.: A1720), DMSO (Cat. No.: D2650), thiazolyl bluet tetrazolium bromide (MTT, Cat. No.: M5655), PMSF protease inhibitor (Cat. No.: P7626), and capsaicin (Cat. No.: M2028) were purchased from Sigma (St. Louis, MO, USA). LY294002 (Cat. No.: S1737), RIPA lysis buffer (Cat. No.: P0013C), nuclear and cytoplasmic protein extraction kit (Cat. No.: P0028), HRP-linked anti-rabbit/mouse secondary antibody (Cat. No.: A0208/A0216), DCFH-DA (Cat. No.: S0033), and DAPI (Cat. No.: C1005) were obtained from Beyotime Company (Shanghai, China). The lactate dehydrogenase (LDH) kit (Cat. No.: 11644793001) and Transcriptor First Strand cDNA Synthesis Kit (Cat. No.: 04379012001) were purchased from Roche Applied Science (Penzberg, Germany). Capsazepine (Cat. No.: HY15640) was purchased from MCE (NJ, USA). Fluo-4/AM (Cat. No.: F14201) was purchased from Invitrogen (San Diego, CA, USA). SDS-polyacrylamide gel (Cat. No.: 1610172) and PVDF membrane (Cat. No.: 1620177) were purchased from Bio-Rad (Hercules, CA, USA). ECL Select Western Blotting Detection Reagent kit (Cat. No.: 12644055) was purchased from GE Healthcare (Chicago, IL, USA). Antibodies against A*β* 1-40 (Cat. No.: 12990), A*β* 1-42 (Cat. No.: 14974), PSEN1 (Cat. No.: 5643), PSEN2 (Cat. No.: 9979), p-CaMKII (Thr 286, Cat. No.:12716), CaMKII (Cat. No.: 11945), CaMKIV (Cat. No.: 4032), Nrf2 (Cat. No.: 12721), Keap1 (Cat. No.: 8047), NQO1 (Cat. No.: 62262), HO-1 (Cat. No.: 43966), p-Akt (Ser 473, Cat. No.: 4060), Akt (Cat. No.: 4691), p-Gsk3*β* (Ser 9, Cat. No.: 5558), Gsk3*β* (Cat. No.: 9315), GAPDH (Cat. No.: 2118), and lamin B (Cat. No.: 13435) and Alexa-Fluor-488 conjugated anti-rabbit IgG secondary antibody (Cat. No.: 4412) were purchased from Cell Signaling Technology (Danvers, MA, USA). Antibodies against APP (Cat. No.: ab32136), BACE1 (Cat. No.: ab183612), and TRPV1 (Cat. No.: ab6166) were purchased from Abcam (Cambridge, UK). Antibody against p-CaMKIV (Thr 196, Cat. No.: PA5-37833), protease inhibitor cocktail (Cat. No.: 78446), and BCA Protein Assay Reagent (Cat. No.: 23225) were purchased from Thermo Fisher Scientific (Waltham, MA, USA). Antibody against Nrf2 (for immunofluorescence assay; Cat. No.: 66504) was purchased from Proteintech (Rosemont, IL, USA). RNAprep Pure Cell/Bacteria Kit (Cat. No.: 4992235) was purchased from TIANGEN (Beijing, China). SYBR® Premix Ex Taq™ II Kit (Cat. No.: RR82LR) was purchased from TaKaRa (Shiga, Japan).

### 2.2. Cell Culture and Treatment

Wild-type N2a (N2a/WT) cells were obtained from the American Type Culture Collection (ATCC, Manassas, VA, USA). N2a cells stably expressing human Swedish mutant APP695 gene (N2a/APP, also called N2S) were gifted from Prof. Jia-Hong Lu (University of Macau, Macao, China) [36]. Cells were maintained in DMEM medium containing 10% (v/v) FBS and 1% (v/v) PS and incubated in a humidified atmosphere of 5% CO_2_ at 37°C. G418 (1%, v/v) was dissolved in culture medium to select transfected N2a/APP cells.

N2a/WT cells were used as a control, and N2a/APP cells were used as a cell model of AD. After seeding for 24 h, cells were treated with DMSO or PcActx peptide at different concentrations (5 or 10 *μ*m) for 24 h. In the following experiments, cells were exposed to LY294002 (10 *μ*m) for 2 h prior to 24 h PcActx peptide incubation. PcActx peptide was dissolved in dd-H_2_O, and LY294002 was dissolved in DMSO, as a stock solution (10 mM). Both solutions were stored at -20°C for bioactive assay.

### 2.3. Cell Viability and Cytotoxicity Assay

Cell viability and cytotoxicity were measured by MTT and LDH assay, respectively. Briefly, N2a/WT and N2a/APP cells were placed in 96-well culture plates (8 × 10^3^ cells/well) for 24 h and subjected to the indicated treatments. The medium was collected for LDH detection according to the manufacturer's protocol, while the remaining cells were incubated with MTT to assess viability. The absorbance at 490 nm for LDH assay and 570 nm for MTT assay were measured using the FlexStation 3 Microplate Reader (Molecular Devices, Sunnyvale, CA, USA).

### 2.4. Calcium Measurement

A calcium marker (Fluo-4/AM) was employed to evaluate the calcium response of the cultured cells. Cells were seeded on a 12-well plate and pretreated with capsazepine (2.5 *μ*M, TRPV1 antagonist) or PcActx peptide (5 and 10 *μ*M) for 4 h. Cells were then stained with 2 *μ*M Fluo-4/AM in the dark at 37°C for 30 min in Tyrode's solution, and excess Fluo-4/AM probe was washed three times with Tyrode's solution. Subsequently, the cells were challenged with 250 *μ*M capsaicin (TRPV1 agonist), and the calcium response (FITC) was immediately estimated using the Cell^R^ imaging system of an IX81 florescence microscope (Olympus Corp., Tokyo, Japan).

### 2.5. Western Blot Analysis

The whole-cell proteins were extracted by ice-cold RIPA lysis buffer using protease and phosphatase inhibitor cocktail. Subcellular fractionations were prepared using nuclear and cytoplasmic protein extraction kits. The total protein concentration was measured by BCA protein assay reagent. Aliquot protein samples (30 *μ*g) were electrophoresed in 10% or 12% SDS-polyacrylamide gel and transferred onto PVDF membranes. Membranes were blocked by 5% nonfat milk for 1 h and then incubated with specific primary antibodies (1 : 1000 dilution) overnight at 4°C. The blots were subsequently incubated with HRP-conjugated anti-rabbit/mouse secondary antibodies (1 : 2000 dilution) and visualized with an ECL kit using the Bio-Rad ChemiDoc XRS Imaging System. The densitometry of the bands was quantified via the ImageJ software (NIH, Bethesda, MD, USA).

### 2.6. Quantitative Real-Time PCR

Total RNA and cDNA from N2a/WT and N2a/APP cells were prepared using the RNAprep Pure Cell/Bacteria Kit and Transcriptor First Strand cDNA Synthesis Kit, respectively, following the standard protocol. RT-PCR was conducted using SYBR® Premix Ex Taq™ II on the Vii7 Real-Time PCR System (Thermo Fisher Scientific) according to the manufacturer's instructions. The mRNA level was calculated through the 2^−*ΔΔ*CT^ method after normalization to *GAPDH*. The primer sequences used are listed in Table 1.

### 2.7. ROS Detection

Intracellular ROS levels were estimated by the fluorescent probe, DCFH-DA. After treating the cells with PcActx peptide for 24 h, they were incubated with DCFH-DA (10 *μ*M, diluted in DMEM) for 30 min at 37°C in the dark. Excess DCFH-DA was washed off by PBS. The fluorescence intensity (FITC-A) of each group was monitored using the BD Accuri C6 Flow Cytometer (BD Biosciences, Franklin Lakes, NJ, USA). For each sample, at least 10,000 events were recorded, and the obtained data were quantitatively analyzed by the FlowJo software (FlowJo LLC, Ashland, OR, USA).

### 2.8. Immunofluorescence Assay

Cells (5 × 10^4^ cells/well) were seeded in a 24-well plate for 24 h and treated with PcActx peptide for another 24 h. After that, they were fixed by 3.7% PFA for 15 min and permeabilized by 0.3% Triton X-100 (diluted in PBS) for 20 min. Then, the cells were blocked with 2% BSA for 1 h and subsequently incubated with anti-Nrf2 primary antibody (1 : 500) overnight at 4°C. Cells from all group were further incubated with Alexa-Fluor-488 conjugated anti-rabbit IgG secondary antibody (1 : 1000) for 1 h in the dark at room temperature. DAPI staining was applied for nuclei observation. The samples were imaged using the cellSens imaging system of an IX73 florescence microscope (Olympus Corp.).

### 2.9. Statistical Analysis

All statistical analyses were performed using the GraphPad Prism software (ver. 8.0; GraphPad Software, Inc., San Diego, CA, United States). Results are presented as means ± standard deviation (SD). The normality and homogeneity of variance of the data were assessed using the D'Agostino-Pearson and Bartlett' tests, respectively. All data were found to be normally distributed and homogeneous, and one-way ANOVA was used for the analysis, followed by Dunnett's test for multiple comparisons. *p* < 0.05 was considered as statistically significant.

## 3. Results

### 3.1. PcActx Peptide Showed No Obvious Cytotoxicity in N2a/WT or N2a/APP Cells

**Figure 1:**
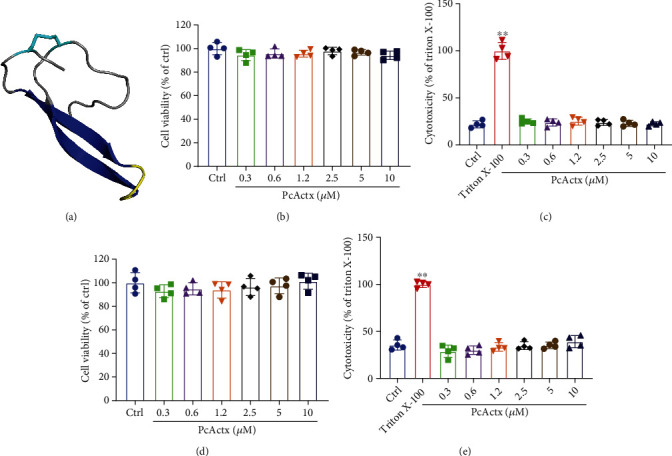
Cell viability of and cytotoxicity in N2a/WT and N2a/APP cells after 24 h of PcActx peptide treatment. MTT and LDH assay were employed to detect cell viability and cytotoxicity, respectively. (a) Homology model structure of PcActx peptide. Molecules are displayed as cartoons and colored according to the secondary structure: *α*-helix in purple, *β*-sheet in blue, *β*-turn in yellow, coil in gray, and disulfide bond in cyan. (b, c) Viability of N2a/WT cells and cytotoxic effects of PcActx peptide. (d, e) Viability of N2a/APP cells and cytotoxic effects of PcActx peptide. Data are expressed as the mean ± SD of four independent experiments, in duplicate or triplicate. ^∗∗^*p* < 0.01 vs. Ctrl group.

The viability and cytotoxicity in N2a/WT and N2a/APP cells after 24 h exposure to PcActx peptide (Figure 1(a)) were firstly detected by MTT and LDH assay. The MTT results (Figures 1(b) and 1(d)) indicated that treatment with PcActx peptide at dosages of 0.3-10 *μ*M did not impact the viability of N2a/WT or N2a/APP cells. Similar results were obtained via LDH assay (Figures 1(c) and 1(e)): PcActx peptide did not cause obvious cytotoxicity in N2a/WT or N2a/APP cells. Moreover, precipitation was observed in the cultured medium when the concentrations were >10 *μ*M. Therefore, N2a/WT and N2a/APP cells were treated with PcActx peptide for 24 h (maximum concentration of 10 *μ*M) in further investigations.

### 3.2. PcActx Peptide Attenuated APP Processing and Amyloid Protein Accumulation in N2a/APP Cells

**Figure 2:**
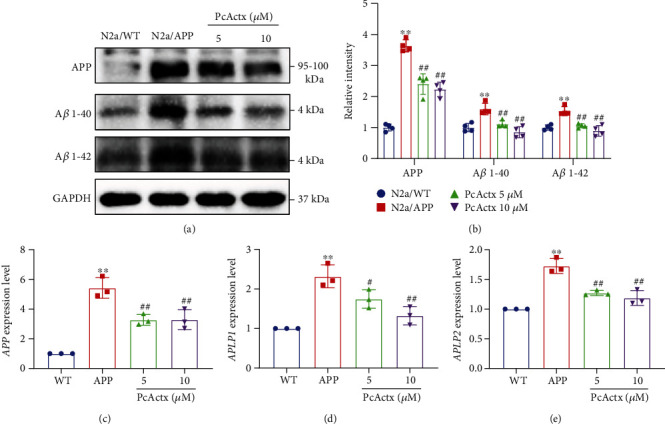
PcActx peptide inhibited the accumulation of APP, A*β*, and APLP proteins. (a, b) The levels of APP, A*β* 1-40, and A*β* 1-42 were determined by Western blot analysis. (c–e) The expression of *APP*, *APLP-1*, and *APLP-2* was detected by RT-PCR. Data are expressed as the mean ± SD of three or four independent experiments, in duplicate or triplicate. ^∗^*p* < 0.05 vs. N2a/WT group, ^∗∗^*p* < 0.01 vs. N2a/WT group, ^#^*p* < 0.05 vs. N2a/APP group, and ^##^*p* < 0.01 vs. N2a/APP group.

A*β* protein (including A*β* 1-40/42) is the principal hallmark of AD and is generated by the alternative splicing of APP designated *β*-secretase (BACE1) and the *γ*-secretase complex [8, 9]. Presenilins (PSEN1 and PSEN2) are the major constituents of *γ*-secretase and mediate APP processing [37]. In addition, amyloid precursor-like proteins (APLP1 and APLP2) are highly homologous to APP, which belong to the APP family. Accumulating evidence indicates that APLPs accumulate in SP in the AD brain, suggesting that APLPs might contribute to AD-associated neurodegeneration [38–40]. In comparison with N2a/WT cells (Figures 2(a) and 2(b)), the protein levels of APP, A*β* 1-40, and A*β* 1-42 were higher in N2a/APP cells, where N2a cells were engineered to express the recombinant human APP695 gene. However, treatment with PcActx peptide for 24 h significantly decreased the levels of APP, A*β* 1-40, and A*β* 1-42 in N2a/APP cells. Consistent with the Western blot results, PcActx peptide obviously reduced the mRNA levels of *APP*, *APLP1*, and *APLP2* in N2a/APP cells (Figures 2(c)–2(e)).

**Figure 3:**
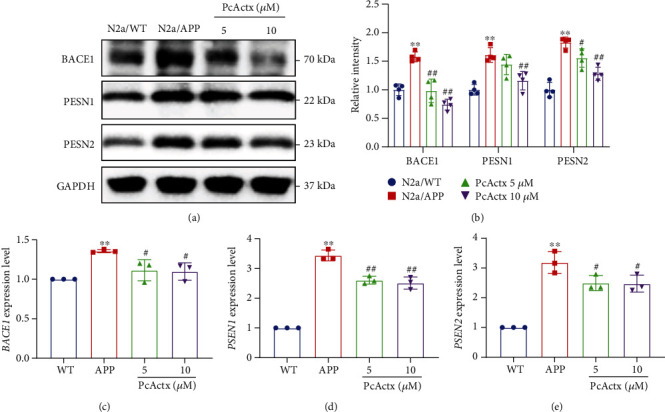
PcActx peptide ameliorated the *β*- and *γ*-secretase-dependent cleavage of APP. (a, b) Protein levels of BACE1, PSEN1, and PSEN2 were determined by Western blot analysis. (c–e) mRNA expression of *BACE1*, *PSEN1*, and *PSEN2* was detected by RT-PCR. Data are expressed as the mean ± SD of three or four independent experiments, in duplicate or triplicate. ^∗^*p* < 0.05 vs. N2a/WT group, ^∗∗^*p* < 0.01 vs. N2a/WT group, ^#^*p* < 0.05 vs. N2a/APP group, and ^##^*p* < 0.01 vs. N2a/APP group.

To further confirm the protective effect of PcActx peptides in the N2a/APP cell model of AD, the protein and mRNA levels of proteolytic enzymes implicated in APP processes were determined by Western blot and RT-PCR, respectively. As depicted in Figures 3(a) and 3(b), the protein levels of BACE1, PSEN1, and PSEN2 were markedly downregulated following 24 h PcActx peptide treatment. Moreover, both 5 and 10 *μ*M PcActx peptide markedly inhibited the gene expression of *BACE1*, *PSEN1*, and *PSEN2*, in parallel to the N2a/APP group without PcActx peptide treatment (Figures 3(c)–3(e)). These results indicated that PcActx peptide could interfere with APP processing and inhibited abnormal amyloid protein production.

### 3.3. PcActx Peptide Inhibited TRPV1-Dependent Calcium Accumulation and TRPV1/CaMK Activation in N2a/APP Cell

**Figure 4:**
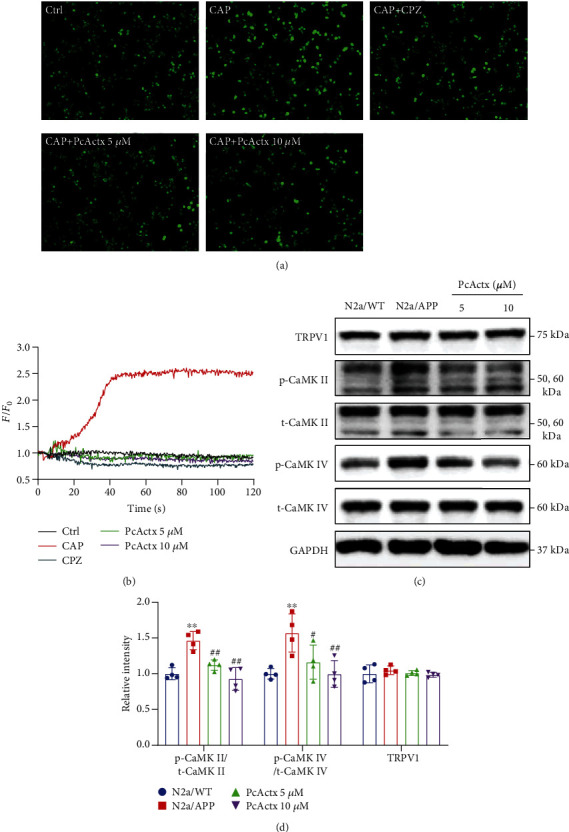
PcActx peptide inhibited TRPV1-dependent calcium accumulation and TRPV1/CaMK activation. (a) Representative fluorescence images of the intracellular calcium response of N2a/APP cells (CAP: capsaicin and CPZ: capsazepine). (b) Time-dependent calcium response revealed by the green fluorescence intensity in representative plots (*n* = 5) before (*F*_0_) and after capsaicin addition (*F*). (c, d) Protein expression levels of TRPV1 and total and phosphorylated CaMKII and CaMKIV were determined by Western blot analysis. Data are expressed as the mean ± SD of three or four independent experiments, in duplicate or triplicate. ^∗^*p* < 0.05 vs. N2a/WT group, ^∗∗^*p* < 0.01 vs. N2a/WT group, ^#^*p* < 0.05 vs. N2a/APP group, and ^##^*p* < 0.01 vs. N2a/APP group.

The TRPV1 channel plays a pivotal role in maintaining calcium homeostasis, which subsequently mediates mitochondrial and synaptic function, as well as neuronal survival and development [41]. In our previous study, PcActx peptide efficaciously suppressed the calcium influx evoked by capsaicin, which is a TRPV1 agonist, in HEK293/hTRPV1 cells, indicating that PcActx peptide is a potential TRPV1 channel blocker [29]. In the present study, we also investigated the effect of PcActx peptide on the TRPV1-mediated calcium response in N2a/APP cells. As can be seen in Figures 4(a) and 4(b), 250 *μ*M capsaicin induced dramatic calcium influx into the N2a/APP cell. However, the capsaicin-stimulated calcium influx was notably counteracted by pretreatment with PcActx peptide and capsazepine (a TRPV1 channel antagonist) for 4 h. Furthermore, PcActx peptide obviously decreased the phosphorylation of CaMKII (Thr 286) and CaMKIV (Thr 196) (Figures 4(c) and 4(d)) in a dose-dependent manner, which can be aberrantly activated by intracellular calcium accumulation [42]. In contrast, PcActx peptide did not affect TRPV1 protein expression (Figures 4(c) and 4(d)). The above results demonstrated that PcActx peptide can modulate the calcium response through TRPV1 channel inactivation, without affecting the TRPV1 protein expression level.

### 3.4. PcActx Peptide Activated the Nrf2/Keap1 Pathway and Promoted Nrf2 Nuclear Translocation in N2a/APP Cells

**Figure 5:**
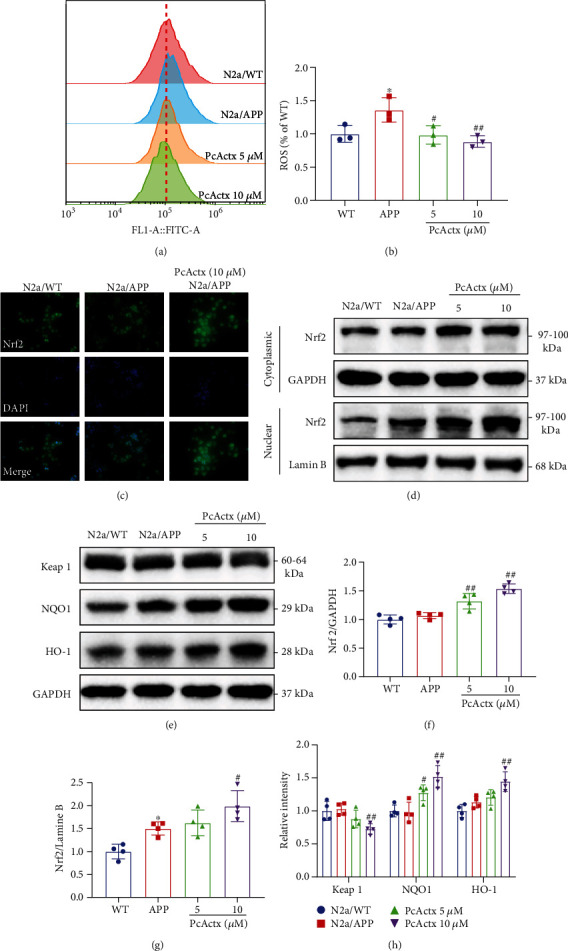
PcActx peptide inhibited oxidative stress and induced Nrf2/Keap1 activation in N2a/APP cells. (a, b) Flow cytometry analysis of the effect of PcActx peptide on ROS production. (c) Representative immunofluorescence images of Nrf2 nuclear translocation in each group. (d–h) Levels of Nrf2, Keap1, NQO1, and HO-1 were determined by Western blot analysis. Data are expressed as the mean ± SD of three or four independent experiments, in duplicate or triplicate. ^∗^*p* < 0.05 vs. N2a/WT group, ^∗∗^*p* < 0.01 vs. N2a/WT group, ^#^*p* < 0.05 vs. N2a/APP group, and ^##^*p* < 0.01 vs. N2a/APP group.

Since neurons are highly susceptible to oxidative damage, oxidative stress is commonly implicated in AD etiology. Activation of the Nrf2 pathway and its subsequent upregulation of the antioxidative system trigger protective mechanisms against oxidative insult [15]. Flow cytometry analysis (Figures 5(a) and 5(b)) showed that PcActx peptide at concentrations of 5 and 10 *μ*M markedly attenuated the excessive production of ROS in N2a/APP cells. As shown by the Western blot results, PcActx peptide, especially at 10 *μ*M, remarkably enhanced cytoplasmic and nuclear Nrf2 expression (Figures 5(d), 5(f), and 5(g)). The elevation of nuclear Nrf2 FITC fluorescence induced by PcActx peptide revealed by immunofluorescence assay (Figure 5(c)) also supported these observations, where PcActx peptide obviously facilitated the nuclear localization of Nrf2 in N2a/APP cells. In addition, PcActx peptide effectively decreased Keap1 expression and increased the levels of NQO1 and HO-1 in N2a/APP cells (Figures 5(e) and 5(h)). These data provided evidence that the activation of Nrf2/Keap1 pathways might be involved in the antioxidative effects of PcActx peptide in N2a/APP cells.

### 3.5. PcActx-Induced Nrf2/Keap1 Activation and A*β* Downregulation Might Be Mediated by the Akt/Gsk3*β* Pathway

**Figure 6:**
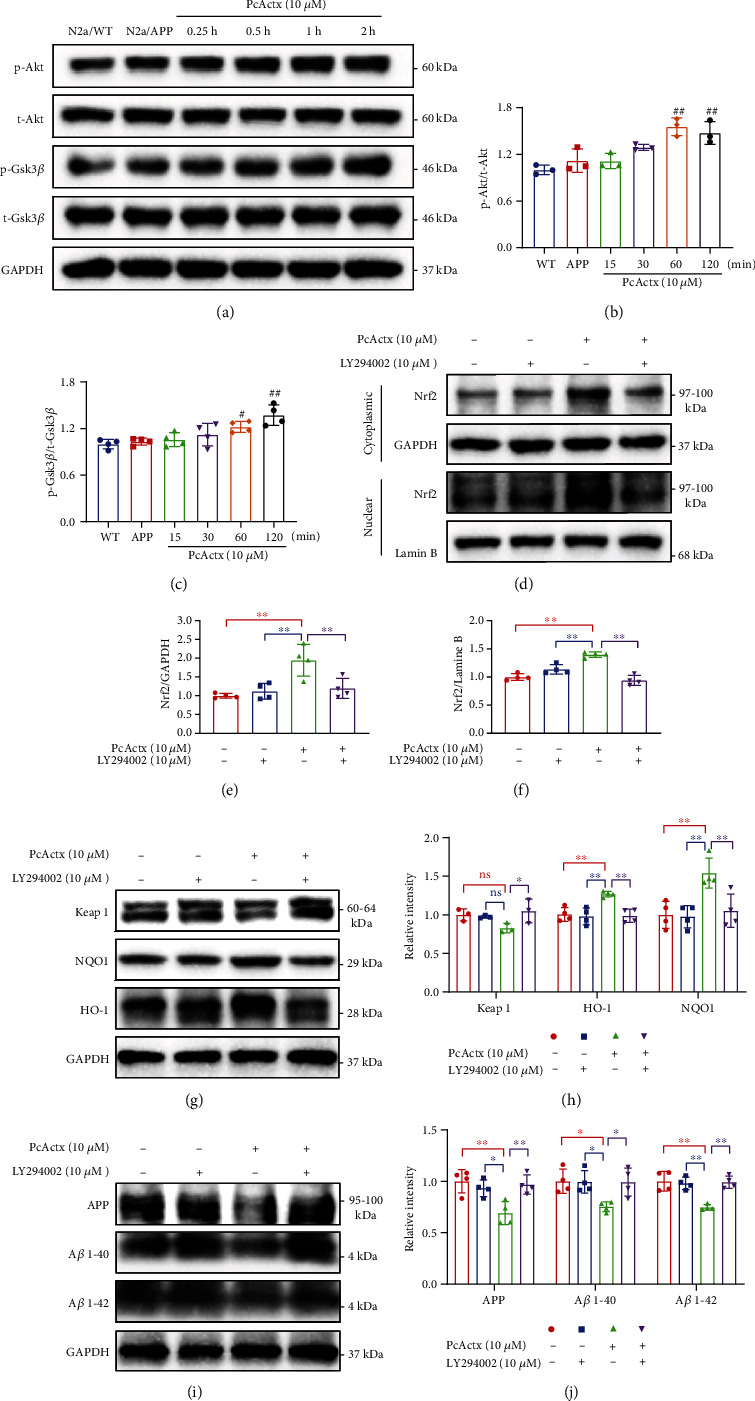
PcActx-induced Nrf2 activation and A*β* downregulation were mediated by the Akt/Gsk3*β* pathway. (a–c) PcActx peptide promoted the phosphorylation of Akt (Ser 473) and Gsk3*β* (Ser 9). (d–h) An Akt inhibitor suppressed the PcActx-induced upregulation of Nrf2, NQO1, and HO-1, as well as the downregulation of Keap1. (i, j**)** The reductions of APP, A*β* 1-40, and A*β*1-42 were counteracted by pharmacological inhibition of Akt/Gsk3*β* signaling. Data are expressed as the mean ± SD of three or four independent experiments, in triplicate. ^#^*p* < 0.05 vs. N2a/APP group and ^##^*p* < 0.01 vs. N2a/APP group. ^∗^*p* < 0.05 vs. PcActx-treated group and ^∗∗^*p* < 0.01 vs. PcActx-treated group.

Akt activation inhibits Gsk3*β* activity, thereby promoting Nrf2 activation and nuclear translocation [15]. Western blot results showed that treatment with PcActx peptide obviously increased the phosphorylation of Akt at Ser 473 (active) and Gsk3*β* at Ser 9 (inactive) within 60 min, without affecting the total levels of Akt or GSK3*β* (Figures 6(a)–6(c)). To determine whether this signaling is involved in the neuroprotective effect of PcActx peptide, N2a/APP cells were preincubated with an Akt inhibitor (LY294002) for 2 h prior to 24 h PcActx treatment. We found that the Akt inhibitor significantly counteracted the PcActx-induced upregulation of cytoplasmic and nuclear Nrf2 expression (Figures 6(d)–6(f)). Furthermore, compared to the PcActx-treated group, the enhanced expression of NQO1 and HO-1 seen in N2a/APP cells was also clearly reversed by the Akt inhibitor (Figures 6(g) and 6(h)). Conversely, the PcActx-mediated inhibitory effects on Keap1 were abolished by the Akt inhibitor (Figures 6(g) and 6(h)). Consistent results were observed in in terms of the expression of APP, A*β* 1-40, and A*β* 1-42 (Figures 6(i) and 6(j)). These findings demonstrated that PcActx-induced Nrf2/Keap1 activation and amyloid downregulation were mediated by Akt/Gsk3*β* signaling.

## 4. Discussion

Bioactive peptide toxins derived from cnidarians may confer neuroprotective effects due to their interaction with virous ion channels [43]. In our pervious study, PcShK and PcKuz peptides from *P. caribaeorum* exhibited potential as voltage-gated potassium channel (K_V_) inhibitors and significantly suppressed 6-OHDA-induced neurotoxicity in a Parkinson's disease (PD) model [27, 44]. PpV*α* peptide from *P. variabilis* was shown to interact with the voltage-gated sodium (Na_V_) channel and displayed neuroprotective effects against PTZ-induced epileptic seizure and 6-OHDA-induced neurotoxicity [45]. Interestingly, among all of the bioactive peptides from Cnidarian identified in this and previous studies, to best of our knowledge, PcActx peptide obtained from the transcriptome of *P. caribaeorum* is the first TRPV1 inhibitor identified and also exhibited antiepileptic activity by reducing ROS production and regulating calcium and GABAergic-glutamatergic signaling; thus, PcActx peptide might exert neuroprotective effects [29]. In the current study, we further demonstrated the neuroprotective potential of PcActx peptide and the underlying mechanism, from a new perspective by employing the N2a/APP cell line as an AD model.

The presence of extracellular amyloid plaques is an important histopathological characteristic in AD. Oligomeric species of A*β* (oA*β*) can induce synaptic dysfunction, thereby contributing to the learning and memory impairment seen during AD progression [46, 47]. Additionally, A*β* aggregation may also cause the calcium homeostasis disruption, mitochondria dysfunction, and oxidative stress, eventually leading to neuronal cell death [48]. In the present study, we employed the stably transfected N2a cell with human Swedish mutant APP, which is a well-characterized cell model of AD, to evaluate the effect of PcActx peptide on amyloid neuropathology. PcActx peptide obviously reduced the protein levels of APP, A*β* 1-40, and A*β* 1-42 (Figures 2(a) and 2(b)). Similar results were observed in terms of the mRNA expression of *APP*, *APLP1*, and *APLP2* (Figures 2(c)–2(e)). APLPs are homologues of APP, which play an important role in synaptic plasticity, neurite outgrowth, neural cell migration, and neural network function [49, 50]. APLP2 can compete with APP and be processed by *β*- and *γ*-secretases, which may suppress A*β* production [50]. However, equivocal results were also found, where APLPs are highly expressed along with SP in the AD brain, suggesting that APLPs might play a role in AD pathogenesis [38–40]. In addition, the protein and mRNA levels of BACE1, PSEN1, and PSEN2 were significantly decreased by PcActx treatment (Figure 3). BACE1 and PSENs are responsible for the N-terminal and C-terminal cleavage of APP, respectively, and promote A*β* aggregation [37]. Furthermore, there is strong evidence that mutations in *PSENs* related to APP processing participate in early onset familial AD. These mutations could increase toxic A*β* production and aggregation, especially of A*β* 42, thus accelerating disease progression [51, 52]. These results suggested that one possible approach for preventing A*β* protein generation using PcActx peptide might be inhibition of *β*- and *γ*-secretase-dependent cleavage of APP.

TRPV1 is a calcium permeable channel expressed not only in the plasma membrane, but also in the endoplasmic reticulum [53]. It acts as a store-operated calcium channel and receptor-operated calcium channel and is responsible for maintaining intercellular calcium homeostasis [30]. Abnormal activation or dysregulation of TRPV1 could induce intracellular calcium overload and thus disrupt calcium homeostasis. Activation of the TRPV1 channel by capsaicinoids has been reported to increase A*β* production through effects on APP processing [34]. In turn, A*β* aggregation further disrupts the calcium-mediated channel, including the TRPV1 channel, and triggers calcium overload, subsequently exacerbating oxidative stress and mitochondrial dysfunction and finally inducing synaptic dysfunction and apoptosis [54–56]. PcActx peptide was shown to be a TRPV1 antagonist in our recent study, as evidenced by the fact that PcActx peptide markedly inhibited the capsaicin-stimulated calcium response in HEK293/hTRPV1 cells [29]. In the present study, a similar mode of action was observed in N2a/APP cells, demonstrating that PcActx peptide can mediate the calcium response through the TRPV1channel (Figures 4(a) and 4(b). Meanwhile, PcActx peptide prominently attenuated the phosphorylation of CaMKII and CaMKIV, which are calcium-mediated proteins, in N2a/APP cells (Figures 4(c) and 4(d)). CaMKII and CaMKIV are activated by the integration of calcium/calmodulin and undergo autophosphorylation [42]. CaMKII is mainly distributed postsynaptically; aberrant activity thereof can cause synaptic dysfunction, leading to the cognitive deficits seen during AD progression [57]. Moreover, the expression and phosphorylation of CaMKIV are elevated by A*β* peptide treatment of PC12 cells [58]. CaMKIV also functions to regulate calcium-dependent gene transcription, such as that involving cAMP response element binding (CREB) protein [42]. hTau accumulation suppresses CaMKIV/CREB signaling in the nucleus and subsequently impairs synapse and memory; however, phosphorylation of CaMKIV was significantly increased in total lysates [59]. In addition, both CaMKII and CaMKIV are tau kinases, the hyperphosphorylation of which can trigger tau phosphorylation and promote NFT aggregation [57, 60]. Accumulating evidence indicates that abnormal activation of CaMKs is involved in ROS generation and oxidative damage. Inhibition of CaMKs could prevent these effects by improving antioxidant defense (e.g., via Nrf2 activation) [61–64].

Since oxidative stress can be considered as both a cause and consequence of calcium dysregulation, its role in AD pathogenesis should not be overlooked [11]. Maintaining calcium homeostasis in turn stabilizes cellular redox homeostasis in the context of oxidative damage. Nrf2 is an antioxidant transcription factor modulating endogenous antioxidant defense systems. In response to oxidative stress, Nrf2 dissociates Keap1 in the cytosol followed by nuclear translocation and binds with ARE to trigger the transcription of a series of antioxidative genes [14]. The calcium stabilizer, ITH14001, exerted a neuroprotective effect through activation of the Nrf2 pathway [65]. In the current study, PcActx peptide notably suppressed ROS overproduction (Figures 5(a) and 5(b)), which could damage various cellular biomolecules such as lipids, proteins, and nucleic acids (RNA and DNA) [66]. Next, we observed that PcActx peptide obviously increased the cytoplasmic and nuclear Nrf2 accumulation and favorably activated downstream antioxidative enzymes including HO-1 and NQO1 (Figures 5(c)–5(h)). Conversely, the Keap1 level was markedly reduced by treatment with PcActx peptide (Figures 5(e) and 5(h)). These data indicated that the downstream mechanism underlying the antioxidative effect of PcActx peptide might involve activation of the Nrf2/Keap1 pathway.

Activation of the Nrf2 pathway is not only directly modulated by the Nrf2-Keap1 complex but also indirectly regulated by kinases-mediated phosphorylation. Several proteins and pathways, such as Akt/Gsk3*β*, mitogen-activated protein kinase (MAPK), and protein kinase C (PKC), are involved in the induction of the Nrf2 pathway [15]. Gsk3*β* can phosphorylate Nrf2 in the Neh6 region, forming a recognition motif for *β*-transducin repeat containing E3 ubiquitin protein ligase (*β*-TrCP) and favoring Nrf2 ubiquitination and subsequent degradation [67]. Gsk3*β* is a downstream protein of Akt, and its inactivation via phosphorylation at Ser 9 is dependent on the activation of Akt [68]. Accordingly, Nrf2 accumulation in the nucleus may partially depend on the activation of Akt and subsequent inactivation of Gsk3*β*. Recent studies found that inhibition of Gsk3*β* activity alleviated cognitive deficits and activated the Nrf2 pathway in SAMP8 mice [69]. Huang *et al*. reported that calcium influx via the TRPV1 channel was responsible for UVB-induced Gsk3*β* activation, which stimulated the phosphorylation and ubiquitination of Nrf2 [35]. Furthermore, active Gsk3*β* can induce tau phosphorylation and aggregation to form NFTs [70]. In the present research, PcActx peptide obviously increased the phosphorylation of Akt at Ser 473 (active) and Gsk-3*β* at Ser 9 (inactive) within 60 min (Figures 6(a)–6(c)). An Akt inhibitor (LY294002) was used to further determine the specific role of the Akt/Gsk3*β* pathway in PcActx-mediated Nrf2 activation and neuroprotection in N2a/APP cells. Inhibition of Akt/Gsk3*β* signaling efficaciously suppressed the PcActx-induced upregulation of Nrf2, HO-1, and NQO1, as well as the downregulation of Keap1 (Figures 6(d)–6(h)). Moreover, the Akt inhibitor clearly counteracted PcActx-mediated inhibitory effects on the expression of APP, A*β* 1-40, and A*β* 1-42 (Figures 6(i) and 6(j)). On the basis of these results, we inferred that the PcActx peptide alleviated A*β* neuropathology via Akt/GSK3*β*-mediated Nrf2 activation in N2a/APP cells.

In the last two decades, peptide and protein drugs have shown great promise for the treatment of a wide variety of neurodegenerative diseases [19–22]. However, a formidable hurdle to drug development for neurological disorders is the BBB, which controls the entry of agents into the brain. To overcome this barrier, multiple approaches have been developed to deliver neurotherapeutic agents to the brain. Receptor-, adsorptive-, and carrier-mediated transport systems have been introduced in recent years for delivering neuroprotective proteins and peptides to the brain [71, 72]. Studies have shown that these noninvasive approaches could increase the permeability of the peptide through the BBB, which can resolve the drug delivery problem [71, 72]. For instance, receptor-meditated transcytosis (RMT) can transport certain proteins (e.g., insulin, transferrin, and leptin) through the BBB via corresponding endogenous receptors [22], which indicates that our peptide, which was predicted to bind with ion channel, could be delivered to the brain through RMT. In our previous studies, two Kunitz-like peptides, PcKuz3 from zoantharian *P. caribaeorum* and ZoaKuz1 mat anemone *Zoanthus natalensis*, interacted with K_V_ channel and suppressed 6-OHDA-induced neuronal loss and behavioral deficits in zebrafish larvae [26, 27]. In another of our previous studies, PcActx peptide containing a Kunitz domain acted on the TRPV1 channel and prevented PTZ-induced epilepsy in zebrafish larvae [29]. Similarly, another Kunitz-type peptide called HCRG21, from sea anemone *Heteractis crispa*, reversed 6-OHDA-induced neurotoxicity *in vitro* [28]. Moreover, Angiopeps, a family of Kunitz domain-derived peptides (especially Angiopep-2), could trigger transcytosis and was transported across the BBB via interaction with low-density lipoprotein-related protein 1 (LRP-1) and serves as a new brain delivery system for neuropharmacological drugs [23–25]. All of the above studies provide evidence that Kunitz-type peptides can potentially cross the BBB. Moreover, at a larger scale, various strategies (such as structural modification and the use of liposomes, nanoparticles, and peptide carrier systems) have been developed to enhance BBB penetration in preclinical and clinical research [22]. Our peptide could cross the BBB via any of methods. Due to the complexity of investigations of the permeability of the BBB to peptides, more experiments are needed to determine precisely how the PcActx peptide can cross the BBB.

## 5. Conclusion

In summary, the PcActx peptide, which is a TRPV1 blocker that could inhibit the capsaicin-induced calcium response in N2a/APP cells, prevented the abnormal accumulation of amyloid proteins by suppressing *β*- and *γ*-secretase-dependent cleavage of APP. Further analysis revealed that PcActx peptide activated the Nrf2 pathway to protect against oxidative insult via Akt/Gsk3*β* signaling. Hence, as a TRPV1 modulator, PcActx peptide exhibited prominent neuroprotective activity in an Alzheimer-like A*β* cell model and is therefore a promising candidate for AD therapy.

## Figures and Tables

**Table 1:** Primers for RT-PCR.

Gene	Primer sequence 5′-3′
*APP*	Forward - TTTGGCACTGCTCCTGCT
	Reverse - CCACAGAACATGGCAATCTG
*APLP1*	Forward - ACACCTGGGTCCCAGTGAAT
	Reverse - GAGAGGACGATGAGGGAGCC
*APLP2*	Forward - CGACGGCACCATGTCAGAC
	Reverse - CAACGAGGCATCACGGC
*BACE1*	Forward - GCAGGGCTACTACGTGGAGA
	Reverse - CAGCACCCACTGCAAAGTTA
*PSEN1*	Forward - TACAAGTACCGCTGCTACAAGTTC
	Reverse - GCACTTCCCCAAGGTAGATATAGG
*PSEN2*	Forward - TACAAGTACCGCTGCTACAAGTTC
	Reverse - GCACTTCCCCAAGGTAGATATAGG
*GAPDH*	Forward - TGCACCACCAACTGCTTAGC
	Reverse - GGCATGGACTGTGGTCATGAG

## Data Availability

The data used to support the findings of this study are available from the corresponding author upon reasonable request.
